# Charleston Almshouse Hospital

**Published:** 1830-05

**Authors:** 


### CHARLESTON ALMSHOUSE HOSPITAL.

### *On the Use of the Pyroligneous Acid in the Treatment of Gangrene, Ulcers, and Fungus Hœmatodes, with an Account of some Cases in which it was successfully employed.* By Thomas Y. Simons, M.D., President of the Medical Society of South Carolina, and Physician to the Almshouse Hospital, Charleston, &c.‡

There are two kinds of pyroligneous acid found in the apothecaries' shops: one is transparent, and, when agitated, shows small crystals floating in it; the other is dark and smoky; both have the empyreumatic odour. The former is the kind I use, and is by far the best.

When I first used this acid, I diluted it with six times its quantity of water; but since I have employed it diluted with equal parts of water, gradually diluting as the sore assumes a healthy appearance, until it becomes as weak as one twenty-fourth. It should always create a smarting sensation. The manner of applying it is to put over the ulcer some lint, which is to be kept constantly wet, and changed two or three times during the day,

‡ American Journal of Med. Sciences.

according to circumstances. The ulcer ultimately assumes red granulations resembling the inside of the pomegranate. If the acid be too strong, it will make it turn white, and assume the appearance of a slough.

Case I. William Smith was brought into the hospital, May 9th, suffering under Mania a potu. After he was relieved of this disease, I observed on the anterior part of his right leg a dark spot occupying about two thirds, where a blister had been applied, as he informed me, previous to his entering the hospital. The commencement of mortification was evident, and I ordered him at first bark poultice, not having at that time the pyroligneous acid in the hospital, and the following constitutional treatment: ℞. Sulph. Quinine gr. iv.; Aq. fontana ℥viij.; Acid Sulphuric gtts. xx.; two table spoonsful to be given every two hours during the day. At night he was given two grains of Opium and five grains of Camphor. He was allowed a pint of porter and a meat diet.

This course was continued for two days, but without checking the gangrene; indeed, it was so rapidly advancing, that several physicians were of opinion that immediate amputation would be necessary.

Having, however, obtained the pyroligneous acid, I resolved to use it first: accordingly, I made free longitudinal and transverse incisions to the full depth of the gangrened portion, and then water and pyroligneous acid, in equal portions, were applied constantly in the manner already described, and the constitutional treatment was continued. In twenty-four hours a line of demarcation was formed, and in twenty-four hours more the gangrenous portion was separating from the healthy part. In seven days the whole of the gangrene was removed, and a healthy surface was presented. The acid, giving pain, was reduced to one sixth, and ultimately to one twelfth; and on the 26th September the patient was dismissed cured. The length of time of healing was produced, I think, from my omitting the acid after healthy granulations were formed, and using the adhesive straps.

Case II. Edward Campbell, from St. John's, Berkely, South Carolina, came into the hospital on the 24th of August. He said that, about Christmas, he had bruised his shin, which he neglected. It was afterwards quacked by some old woman in the parish, until it assumed the character which I shall now describe. There was an extensive sloughing ulcer, deep, irregular, and jagged, extending from the lower portion of the tibia two thirds upwards, exposing a part of the bone which was carious, and the tendon of the extensor longus digitorum pedis. The fetor from the ulcer was so great as to induce me to remove the patient to a place separate from the other inmates of the hospital. My patient was extremely emaciated and hectic; and I observed to the medical gentlemen and students who were present, that I had no hopes of saving the limb, but that it was desirable to place him under constitutional treatment, so that I might improve the conservative principle of the system, (to adopt Sir G. Blane's language,) previous to my amputating the leg, and that I would apply the strongest solution of the acid, merely to correct the fœtor. The treatment was, ℞. Sulph. Quinine gr. vi.; Acid Sulph. gtts. xx.; Aq. fontana ℥viij.; two table-spoonsful every two hours during the day. At night, two grains of opium, to lessen irritation and procure sleep, which he had not enjoyed for some months. The diet was a pint of porter daily and beefsteak.

In two days the fœtor of the ulcer was overcome. In ten days it was much improved, and I took away a large piece of bone which had exfoliated from the tibia. In four days more I removed with the knife a considerable slough of the tendon of the extensor longus digitorum pedis. From this time the ulcer began to improve rapidly, and healthy granulations appeared.

This course was persevered in for some time with continued improvement of the leg, when my patient suffered it to be kept hanging down, causing the blood to determine and stagnate at the ulcer, when an extensive sloughing and gangrene commenced, (the acid having then been omitted,) which continued for three days, until the pure acid (the brown and smoky one having been sent me by the apothecary, which proved inert,) was obtained, which checked its progress in twenty-four hours, and removed it altogether in a week. The patient was made to keep his leg elevated, and the acid was continued until November 7th, at which time the leg had almost healed, and the acid was omitted.

Case III. Charles Belton was brought into the hospital on the 13th of September, suffering from the effects of intemperance. I observed a red suffusion over his left thumb, with considerable tumefaction: he complained of its giving him great pain. I ordered a poultice of milk and bread. This was continued for three days, when the inflammation increased, became more painful and tumefied; a fluctuation was felt as if there was matter, and there appeared to be a disposition to point over the second articulation of the thumb. I made a free incision, when very little matter escaped, but a great quantity of blood. On the next morning, I was informed that upwards of two pounds of blood had come from the wound, although I regarded this quantity as exaggerated. I found, upon examination, the wound had all the appearances of fungus hæmatodes: it spread out on each side of the incision like a mushroom, was fungous, very vascular, and oozing blood at every part. So formidable an appearance in so short a time left little hopes of relief but in removing the diseased part, which remedy is more than equivocal as regards success. It was, however, suggested to me by a medical friend, that, as the pyroligneous acid had proved so valuable and efficacious in the other cases, whether it would not be worthy of a trial in a disease which has generally defied the power of remedial agents. I readily consented, but with no hopes of success. The acid was applied in its strongest form, which in two days checked the hemorrhagic tendency. In fifteen days the fungous character of the wound was subdued, when lunar caustic and adhesive straps were applied, which completed the cure on the 25th of October.

During the prevalence of yellow fever in Charleston, in 1824, I gave the acid, much diluted, internally during the black-vomit stage, but with no benefit. I have no doubt it would prove salutary in putrid sorethroats as a gargle, and it would be worthy of trial in cancer, in neither of which have I yet used it.

I have drawn up these cases and observations from a conscientious conviction that a proper use of the pyroligneous acid will be the cause of saving to many human beings limbs which otherwise would be cut off, and with the anxious hope that its use among surgeons may become general.

